# An Experience with Cannabis Indica

**Published:** 1884-05

**Authors:** Frank Dudley Beane

**Affiliations:** New York City


### An Experience with Cannabis Indica.

By Frank Dudley Beane, A. M., M. D., of New York City.

Having suffered from general neurasthenia for a couple of weeks, and annoyed by pain for which I did not care to take opium or morphia, I took, about 9.45 A M. of March 5th, just seven and a half (7½) minims of "normal liquid" cannabis indica and twenty-two and one-half (22½) minims of "normal liquid" ergot, samples of which had recently been received from the manufacturers, Messrs. Parke, Davis & Co.

About 10.15 A. M., while sitting in my office, I felt a momentary dizziness pass over me, accompanied by a peculiar *lightness* of the whole body. The sensations were repeated. I laid down the medical journal I was reading, arose from my chair, but again came the phenomena, with increased power. I walked up and down the room, experiencing an indefinable nervous depression in addition to the sensations before alluded to. Stamping my foot, I chided myself for allowing this "nervousness" to master my attention. The dizziness increased. I threw myself upon the sofa, thinking to dissipate these feelings by a better supply of blood to the brain. I had before experienced slight vertiginous attacks after prolonged mental work, and was not alarmed. On assuming recumbency the symptoms at once increased, immediately followed by a peculiar and indescribable dread, and general muscular weakness.

Satisfied that I was to experience the peculiar physiological effects of Indian hemp (a vivid description of which I had years ago read, in Dr. H. C. Wood's "Materia Medica," I think), combined with those of ergot, I descended to the dining-room, swallowed half of a wineglassful of port wine, but could not the balance, inasmuch as I felt I had only strength sufficient to climb to the second story, where my wife was. Ascending the two flights of stairs with the greatest difficulty, on account of the lead-like heaviness in my legs, it seemed as though at the next step should fall backward. On entering the room I informed my wife what was to be expected; I was walking about in an aimless, half-dazed way, when she led me to the bed, whereon I threw myself, removing the pillow from under my head. Gradually increasing darkness came on, accompanied by a swimming of my head, a sinking feeling at the præcordial region, a sensation of spasmodic, powerful contractions of all the blood vessels of the body, even of the smallest twigs, and a horrible feeling of impending death. I directed my wife to give me raw brandy, and send for my bottle of liq. atropiæ (Lond.) my object being to antagonize the arterial contraction. Immediately I began moving the right leg up and down, rubbing the foot against the bed-clothes very rapidly (my wife informs me I also slapped the bed with my right arm, for a few moments, but I recollect it not), as the motion seemed to be a relief, in partly distracting my attention from the increasing muscular heaviness, rapid action of the heart, and cold waves, which were passing rapidly and in quick succession over my whole body. The motion was wholly voluntary, as, on the entrance of Dr. Davis, I discontinued it long enough to demonstrate it was under control. I explained to him the state of affairs, advised the giving of atropia as an arterial dilator, and was given brandy and ♏ ij of the solution above-mentioned at his hands. While he was dropping the above my eyelids closed involuntarily, but I did not, even for an instant, lose consciousness. The lids seemed simply to close over the eyes, and I had neither inclination nor power to open them. Next, my breathing became *to me* laborious, as though a great weight were upon my chest interfering with its expansion, as well as an impediment rising from under the sternum toward the larynx.

I should here state that up to just before the arrival of the doctor I had been feeling my pulse, which became so quick and feeble I desisted from further interrogation; being sensible, the knowledge of a failing heart would add nothing to my power of resistance.

Simultaneous with the oppressed breathing (which, by the way, Dr. Davis said became very shallow for a while, requiring his admonition to "breathe more deeply," with which instruction I complied and remember), appeared most intense, and awful coldness in the præcordial region, which seemed actually unbearable the instant preceding the application of (almost) scalding hot water, also applied to my forehead and feet, affording, as it then seemed to me, the greatest relief ever experienced by a suffering mortal!

Suddenly a shock, as it may be best expressed, of motor and sensory paralysis passed through my frame; then I was instantaneously introduced to the next stage. How shall I describe it? To say that like an electric flash an unconsciousness passed over my brain, and that cerebration was instantly awakened to three sensations—one, that I was speeding along like the wind in the utter darkness of a broad, interminable tunnel; another, that my body lay cold in death (upon the bed) at which I was gazing as from some spot in the vale of darkness, while I speculated upon the appearance my corpse presented; the third, a consciousness of what my wife and the doctor were saying. To describe all this but feebly expresses the wonderful triple sensation.

Despite the lightning-like rapidity of these occurrences, my mind had time to soliloquize: "If this be death, 'tis not only devoid of horror and fear, but brings such calm and ease as alone could be vouchsafed mortal by the All-Wise."

The pitch-like darkness gave way to a phosphorescent light, quickly succeeded by the most beautiful and soft lilac shade of misty brightness, lasting sufficiently long for me to exclaim: "Oh! what a beautiful purplish hue!"

Succeeding this came a genuine state of trance, which lasted till 4.30 P. M.

My body seemed to be fashioned from wood. My neck and head were hewn from hickory; my chest and abdomen, of huge proportions, were also wooden, and from my hips my limbs were represented by a log of wood extending an interminable distance along a pathway through woods, the green-leaved trees rising up on either side, and, far above, the clear, blue, beautiful, half-sunny sky seemed to look with benignant glance upon the strange object stretched upon the ground. How queer my carved-from-wood face appeared to my other self, which never for an instant failed to recognize the dual existence, to realize what was transpiring about me in the room, hall, or even the hall below stairs. How extremely funny appeared to my *alter ego* my chest and abdomen, forming a huge barrel! So excruciatingly ludicrous it seemed that, lifting my arm, and crying out: "See how hollow this barrel-stomach of mine is!" I struck out with such force as to make the result anything but agreeable to the part attacked. No sooner had the hand touched my body than the barrel hallucination disappeared; on removing the hand it reappeared. And my arms, how short they seemed, and how immense my hands! The arms seemed thrust out from and attached to openings in the upper part and sides of the barrel, and felt to be no longer than five inches, or half the length of my forearms. The hands seemed like huge boxing gloves!

Henceforward, till I awoke at 4.30 P. M., I remained in a state of the most hilarious exhilation and constant volubility. I was contented and happy beyond description, only wishing for indefinite prolongation of this state. My tongue ran upon much nonsense and every imaginable topic. I asked many questions pertinent to happenings of the past and present, and connected with expected events. Then, again, I would laugh immoderately at some remark (possibly far from mirth-provoking) of the bystanders, or at my own imaginings. (My wife says I cried most piteously for awhile over a matter of the past, present, and future which greatly concerns me, but I do not recollect.) I was ever conscious of all my foolishness, but had no *will-power* to exert control. My hearing reached the highest pitch of acuteness; it could locate the slightest noise even in the lower halls; it caused me to *feel* the presence of members of my family in whatever part of the room or hall they might be. On the entrance of any one my eyelids would fly open, I would raise myself up in bed, and, staring, point my finger as I pronounced the correct name, then fall (or be pushed) back upon the bed, my eyelids closing, and the woody phantasm reappear. Each time (four) I thus opened my eyes all the surroundings appeared natural, even to the snow falling outside, seen through the window, only that I was looking, as it were, through a veil of brilliant ether, for I can in no other words adequately express the appearance. Among others, I had the notion that the extremities of my body were attached to pivots, and that (making the movements with my body sideways) I was swinging to and fro like a baby's cradle. At the same time I realized this was indeed an hallucination.

The atropia I had taken caused such dryness of the fauces, water was given me, and felt as though poured into a long wooden tube, resounding as it touched bottom, instead of into my mouth and throat. The opening of my lips seemed like separating two large blocks of wood (or cork), so immense the labia appeared to be. At 4.30 P. M. I emerged from this state exclaiming: "Why, here I am once more, and everything just as natural as ever. What time is it?" When told, I wondered whence had passed so much time, so short a period it seemed since I went to bed, certainly not more than a quarter of an hour. I was much exhausted, and soon inclined to drowsiness, which I

resisted. *Intense hunger* appeared, but I ate little or nothing. Slept well. Attended to practice the next day, though feeling quite weak and slightly dizzy. On walking the streets, distant objects appeared to be very near, and I imagined myself to be very tall. Both notions disappeared in less than an hour.

Finally, I remember of vomiting once (only) during the reactive (trance) stage, also of relieving my bladder of a goodly quantity of rather light-colored urine. No aphrodisiac effect accompanied or followed the phenomena. In addition to the subjective, above described, the objective symptoms, observed by my wife, were, during the stage of depression, icy coldness of the face, head, chest, upper and lower extremities, blueness of the hands, face of marble-like pallor, under lids puffed in the centre like a ridge (spasm of the orbicularis), pupils widely dilated, eyeballs bloodshot, wild look, no convulsions, general or local. Doctor Davis reported that my pulse at one time could scarcely be detected at the wrist, so feeble and rapid it had become.

*Remarks*—I wish briefly to draw attention to the following points:

1. In practice I have given as many as gtt. xx, and in one case reached gtt. xxxv, at one dose, repeated several times a day for several days, of the fluid extract of cannabis indica, with the best effect upon pelvic pain, and no other disagreeable symptom than slight stiffness of the jaw. I know not the make, but certainly it was not that of Parke, Davis & Co.; have given 3-grain doses of Lazell, Marsh & Gardiner's solid extract every two and three hours for six and eight successive doses without the slightest unpleasant effect, though one dose of gr. ⅓ of the solid extract of unknown (to me) make caused in another (a lady) patient severe stiffness of the jaw, anæmia of the brain (fainting), and nervous trembling, which symptoms disappeared in about an hour.

2. The specific effect of the Indian hemp in this, as in other cases, was first to depress the senso motor system, then to stimulate the ideational, emotional, perceptive, and memorial psychic-centres, the optic, auditory, and pneumogastric nerves, while paralyzing *the will.*

3. I cannot forbear comparing this state of trance with that psychic condition found in the ordinary hysterical paroxysm. In both there is complete subjugation of the *will* to the exalted other psychic functions.

4. Doubtless the serious disturbance of the circulatory system in my experience was largely due to the combination with ergot.

5. Taking the present for a test case, the value of Parke, Davis & Co.'s preparation of haschish as an exhilarant, at least, cannot be questioned.

Those interested in the subject of the physiological action of the drug will find much valuable information and detailed cases in the following works:

1. Pereira: "Elements of Materia Medica," edit. 1860, art. *Cannabis Sativa*, where an interesting case of haschish catalepsy may be found in detail.

2. Stillé: "Therapeutics and Materia Medica," edit. 1860, pp. 81–86; the author referring to one case, among others, that of Dr. John Bell (originally reported in the *Boston Medical and Surgical Journal*, 1857, p. 209), which in some particulars resembles mine.

3. Wood: "Treatise on Therapeutics," edit. 1876, art. *Cannabis Indica*, where he records his own experience, greatly differing from mine.

